# Aquaporin-4 Mediates Permanent Brain Alterations in a Mouse Model of Hypoxia-Aged Hydrocephalus

**DOI:** 10.3390/ijms22189745

**Published:** 2021-09-09

**Authors:** José Luis Trillo-Contreras, Juan José Toledo-Aral, Javier Villadiego, Miriam Echevarría

**Affiliations:** 1 Institute of Biomedicine of Seville-IBiS, University Hospital Virgen del Rocío, CSIC, University of Seville, 41013 Seville, Spain; josetricon@gmail.com (J.L.T.-C.); juanjo@us.es (J.J.T.-A.); 2 Department of Medical Physiology and Biophysics, University of Seville, 41009 Seville, Spain; 3 Network Center for Biomedical Research in Neurodegenerative Diseases (CIBERNED), 28031 Madrid, Spain

**Keywords:** AQP4, astrocytes, hypoxia, hydrocephalus, cerebrospinal fluid, cerebral ventricles

## Abstract

Aquaporin-4 (AQP4) is the principal water channel in the brain being expressed in astrocytes and ependymal cells. AQP4 plays an important role in cerebrospinal fluid (CSF) homeostasis, and alterations in its expression have been associated with hydrocephalus. AQP4 contributes to the development of hydrocephalus by hypoxia in aged mice, reproducing such principal characteristics of the disease. Here, we explore whether these alterations associated with the hydrocephalic state are permanent or can be reverted by reexposure to normoxia. Alterations such as ventriculomegaly, elevated intracranial pressure, and cognitive deficits were reversed, whereas deficits in CSF outflow and ventricular distensibility were not recovered, remaining impaired even one month after reestablishment of normoxia. Interestingly, in AQP4^−/−^ mice, the impairment in CSF drainage and ventricular distensibility was completely reverted by re-normoxia, indicating that AQP4 has a structural role in the chronification of those alterations. Finally, we show that aged mice subjected to two hypoxic episodes experience permanent ventriculomegaly. These data reveal that repetitive hypoxic events in aged cerebral tissue promote the permanent alterations involved in hydrocephalic pathophysiology, which are dependent on AQP4 expression.

## 1. Introduction

Aquaporin 4 (AQP4) is the most abundant and studied aquaporin in the brain; it is expressed preferentially in the endfeet of the perivascular astrocytes that are part of the blood-brain barrier and is abundantly located in the ependymal cells that line the ventricular system [1,2,3]. In recent years, various studies have revealed a prominent role for AQP4 in cerebrospinal fluid production and circulation [4,5,6], describing it as a principal component of the so-called glymphatic system, which facilitates the flow of cerebrospinal fluid (CSF) through the brain parenchyma [6]. Apart from this function as a “strict” water channel, AQP4 is an integral protein of the astrocytes’ cell membrane endfeet, participating in the structural assembly between astrocytes-extracellular matrix and endothelial cells [7]. In particular, AQP4 anchors to the cell membrane in the astrocytes’ endfeet by coexpression with dystroglycan, and it binds intracellularly with alpha-syntrophin, alpha-dystrophin, and dystrobrevin, and with laminin and agrin toward the extracellular side [8,9], forming part of a protein scaffold collectively called the dystroglycan complex [9,10]. All elements of this complex are structurally compromised in the physiological configuration of the blood brain barrier and likely contribute to its permeability or some biophysical properties of the brain tissue like the tissular distensibility [5].

The relevance of AQP4 in response to brain insults such those produced by cerebral ischemia or stroke, in which cytotoxic or vasogenic edema can develop, have been extensively demonstrated [11,12]. Additionally, altered expression or distribution of AQP4 has been associated with hydrocephalus [13]. Hydrocephalus is a neuropathological condition, main characteristic of which is the excessive accumulation of CSF in the brain and often enlargement of the ventricles, which normally leads to an increase in intracranial pressure, which is harmful to brain tissues and is significantly associated with high neurological morbidity and mortality [14]. An increment in the amount of CSF, either due to excessive production or reduced reabsorption, can be associated with altered expression or dysfunction of the AQP4 present at the glia limitans separating the CSF and brain parenchyma. Various animal models, including hydrocephalus with hop gait mice with congenital hydrocephalus [15,16] or hydrocephalus produced by kaolin injection into the cisterna magna of rats [17], have demonstrated an association between hydrocephalus and AQP4 expression. Moreover, an altered distribution of AQP4 in the endfeet of perivascular astrocytes has been reported in patients with idiopathic normal pressure hydrocephalus (iNPH) [18].

In a previous study [19], we showed that hypoxia (Hx) and aging act synergistically to produce hydrocephalus in mice, and three elements decisively contribute to this effect: (i) increased, although disorganized, expression of AQP4 in the brain astrocytes and ependymal cells; (ii) an overload of CSF in the brain ventricles during the hypoxic event; and (iii) a reduction in the CSF evacuation rate and cerebral distensibility. The hydrocephalic situation induced in aged animals exposed to Hx resembles some of the principal physiopathological features observed in patients with iNPH, and led us to suspect that this Hx-aged mouse model would represent an excellent animal model to investigate various aspects related to iNPH pathophysiology. Currently, CSF diversion with ventriculoperitoneal or lumboperitoneal shunt is the principal treatment for iNPH, and although it produces clear symptomatology relief, it is partial and transitory [20,21]. Thus, the aim of the present study was to examine whether the pathophysiological alterations observed during the hypoxic event (ventriculomegaly, slight elevation of intracranial pressure, cognitive impairment, reduced CSF drainage, and decreased ventricular distensibility) persist over time or are recovered after returning the animals to normoxia (Nx). By means of parameters strictly related to CSF homeostasis and cognitive testing, we found that ventriculomegaly, elevated intracranial pressure, and cognitive deficits were reversed by re-normoxia (ReNx). However, although the changes produced in the CSF outflow and ventricular distensibility were permanent in wild type (wt) mice, they were completely reverted by ReNx in AQP4^−/−^ mice, suggesting that AQP4 has a structural role in the chronification of those alterations. Lastly, we investigated how successive hypoxic events could produce a chronic hydrocephalic state, showing that aged mice subjected to two hypoxic episodes experience permanent and irreversible ventriculomegaly. These data reveal, for the first time, that repetitive hypoxic events in aged cerebral tissue promote permanent alterations involved in iNPH pathophysiology, and these anomalies are dependent on AQP4 expression.

## 2. Results

### 2.1. Re-Normoxia Restores CSF Production to Baseline and Reverses Cognitive Decline in Aged wt Mice That Develop Hypoxia-Induced Hydrocephalus

**Figure 1:**
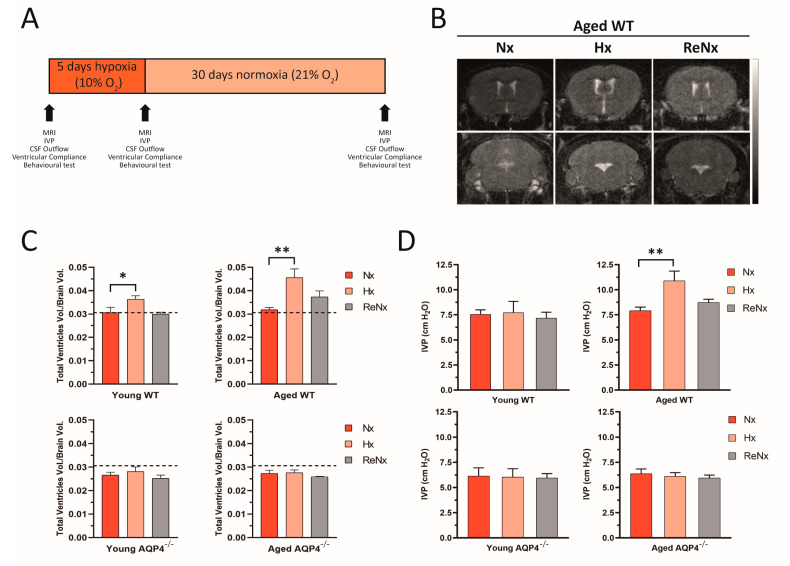
Severe ventriculomegaly in aged wt hypoxic mice is reverted by re-normoxia. (**A**) Diagram showing the experimental protocol followed for each experimental group: wt or AQP4^−/−^ young and aged mice were subjected to normoxia (Nx: 21% O_2_); hypoxia (Hx: 5 days at 10% O_2_); or re-normoxia (ReNx: 30 days at 21% O_2_ after Hx); and a physiological analysis and behavioral test were performed (MRI, IVP, CSF outflow, ventricular compliance, and novel object recognition). The time-point of these experimental analyses is indicated by arrows. (**B**) MRI of brain coronal sections from aged wt mice in Nx, Hx, and ReNx. The gradient scale of T2-weighted MRI indicates the liquid present in the tissue, ranging from bright (high water content) to dark (absence of water). (**C**) Quantification of ventricular volume of each experimental group under the conditions described in (**A**). (**D**) Analysis of IVP measurements recorded in the four study groups, under the conditions mentioned above. Data are presented as mean ± SEM. Number of animals analyzed for each experimental condition: Young-wt, Nx (*n* = 12); Hx (*n* = 11); ReNx (*n* = 6). Aged-wt, Nx (*n* = 5); Hx (*n* = 6); ReNx (*n* = 6). Young-AQP4^−/−^, Nx (*n* = 7); Hx (*n* = 6); ReNx (*n* = 6). Aged-AQP4^−/−^, Nx (*n* = 5); Hx (*n* = 5); ReNx (*n* = 5). * *p* < 0.05; ** *p* < 0.01.

The results obtained in our previous study [19] demonstrate that the disorganized overexpression of AQP4 associated with aging decisively contributes to the initial development of a hydrocephalic condition, which is exacerbated by Hx. To analyze how this hydrocephalus is permanent or could be reverted by returning to Nx, wt and AQP4^−/−^ mice at various ages (young, 2–4 months old; and aged >14 months old) were treated under a variety of oxygenation conditions: Nx, 21% O_2_; Hx, 10% O_2_ for 5 days; and ReNx, 21% O_2_ for 30 days after the hypoxic stimulus. Changes in physiological parameters related to CSF homeostasis (ventricular volume, intraventricular pressure [IVP], CSF outflow, and distensibility of the ventricular system) and associated cognitive function were evaluated under the previously described oxygenation conditions (see diagram in Figure 1A).

Volumetric measurements of the ventricular cavity were analyzed by magnetic resonance imaging (MRI; Figure 1B,C). Hx induced a slight increase in the ventricular volume of young wt mice, which evolved into significant ventriculomegaly when aged wt mice were analyzed. However, when the mice were returned to normoxia (ReNx), the ventricular volume of the young mice was completely recovered, showing values similar to the Nx controls (Figure 1C). In the case of aged wt mice, ReNx treatment produced a partial recovery from severe ventriculomegaly, displaying in these animals a trend toward higher ventricular volume than that of the Nx controls (Figure 1B,C). No changes were observed in the ventricular volume of the AQP4^−/−^ mice, either after Hx or after ReNx, remaining unchanged in both young and aged animals. Consistent with the analysis of the ventricular volume, IVP measurements in young animals showed no variations in any of the three experimental conditions (Nx, Hx, or ReNx) examined. Meanwhile, aged wt mice recovered their IVP value after ReNx, given no statistical differences with the Nx situation were observed at the end of the ReNx period (Figure 1D). In the case of AQP4^−/−^, for both young and aged mice, no changes were observed in IVP after hypoxic treatment; IVP values remained consistently unaltered after ReNx (Figure 1D).

**Figure 2:**
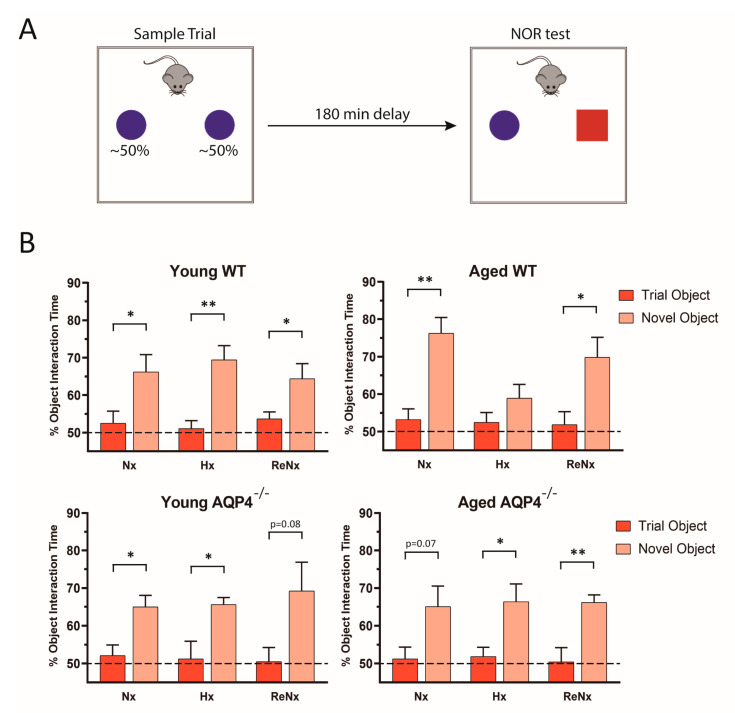
Re-normoxic treatment recovers the cognitive deficit induced by hypoxia in aged wt mice. (**A**) Illustration showing the experimental protocol performed to evaluate cognitive function, by novel object recognition (NOR) test. (**B**)The NOR assessment was performed in wt or AQP4^−/−^, young and aged, mice subjected to normoxia (Nx), hypoxia (Hx), or re-normoxia (ReNx). The analysis represents the interaction time (in percentage, %) the mice spent with the new object compared with the old one, for each experimental group. Dashed lines at 50% of the object interaction time indicates the expected time of interaction for both identical objects during the sample trial. Data are represented as mean ± SEM. Number of animals analyzed for each experimental condition: Young-wt, Nx (*n* = 12); Hx (*n* = 9); ReNx (*n* = 8). Aged-wt, Nx (*n* = 9); Hx (*n* = 10); ReNx (*n* = 9). Young-AQP4^−/−^, Nx (*n* = 9); Hx (*n* = 9); ReNx (*n* = 5). Aged-AQP4^−/−^, Nx (*n* = 6); Hx (*n* = 8); ReNx (*n* = 8). * *p* < 0.05; ** *p* < 0.01.

We also explored whether reversion of the ventriculomegaly observed in the mice subjected to ReNx led them to recover their cognitive function. Using the novel object recognition behavioral test as previously described (Figure 2A), we observed that hypoxic treatment produced significant cognitive impairment only in the aged wt mice (Figure 2B). In these aged wt animals, cognitive function was restored after the re-normoxic period (Figure 2B), a finding that likely indicates that reversion of the severe ventriculomegaly developed by hypoxia allows the recovery of cognitive function. In consonance with the absence of ventriculomegaly, the rest of the experimental animals (young wt, young *AQP4^−/−^* and aged *AQP4^−/−^)* did not present significant cognitive alterations after hypoxic treatment. In these animals the ability to recognize new objects was never affected and remained invariable either after hypoxic treatment or after the re-normoxic period.

### 2.2. CSF Drainage Capacity Remains Impaired after Re-Normoxia in Aged Mice That Develop Hypoxia-Induced Hydrocephalus

**Figure 3:**
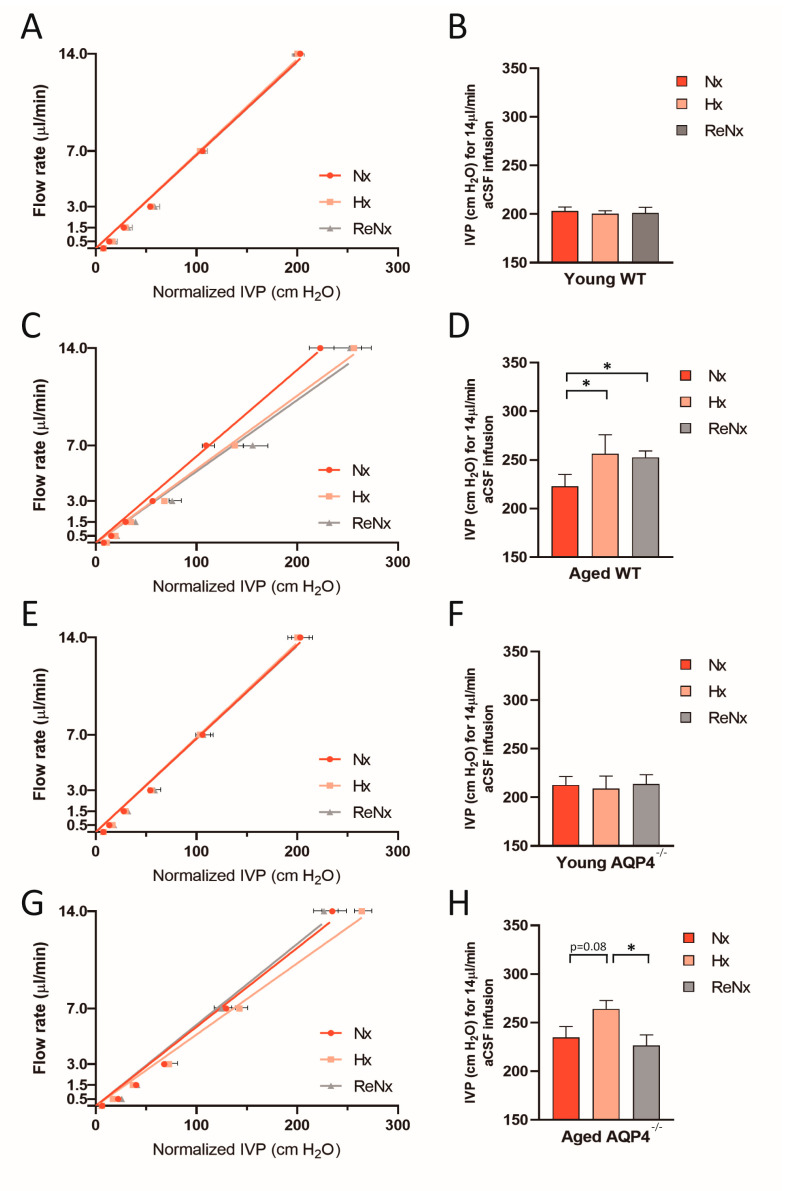
Hypoxia induces a permanent deterioration of CSF drainage capacity in aged wt mice. The figures represent the linear regression obtained for the relationship between the infused artificial CSF rate and the IVP reached in a stationary state, for each experimental group: (**A**,**B**) young wt, (**C**,**D**) aged wt, (**E**,**F**) young AQP4^−/−^, (**G**,**H**) aged AQP4^−/−^, under the experimental conditions indicated (Nx: normoxia; Hx: hypoxia; ReNx: re-normoxia). The normalized IVP was calculated by the subtraction of the basal IVP value (obtained without aCSF infusion) from the IVP values obtained after aCSF infusion at each rate. The bar graphs show the comparative analysis of the IVPs achieved for an infusion rate of 14 µL/min for the different experimental groups. Data are represented as the mean ± SEM. Number of animals analyzed for each experimental condition: Young-wt, Nx (*n* = 12); Hx (*n* = 7); ReNx (*n* = 6). Aged-wt, Nx (*n* = 8); Hx (*n* = 7); ReNx (*n* = 5). Young-AQP4^−/−^, Nx (*n* = 10); Hx (*n* = 8); ReNx (*n* = 8). Aged-AQP4^−/−^, Nx (*n* = 8); Hx (*n* = 6); ReNx (*n* = 8). * *p* < 0.05.

Using a pressure-dependent CSF outflow analysis originally described by Oshio et al. [22], we studied whether the CSF evacuation routes remained permanently reduced after the hypoxic stimulus or returned to a normal state with the reexposure to normoxia. The results shown in Figure 3 again confirm that after Hx treatment the CSF drainage capacity in aged animals, either wt or AQP4^−/−^, was reduced (Figure 3B,D); however, this alteration did not occur in the young mice (Figure 3A,C). After ReNx, the drainage capacity in aged wt mice remains permanently reduced (Figure 3B); meanwhile, in aged AQP4^−/−^ mice, the animals revert to values similar to the ones measured in Nx (Figure 3D). Thus, these results indicate that CSF evacuation capacity is permanently deteriorated in aged wt mice subjected to Hx, which is the unique experimental group that experienced severe ventriculomegaly, and the presence of AQP4 is essential to this process.

### 2.3. Diminished Ventricular Distensibility Is Not Recovered after Re-Normoxia in the Aged wt Mice

**Figure 4:**
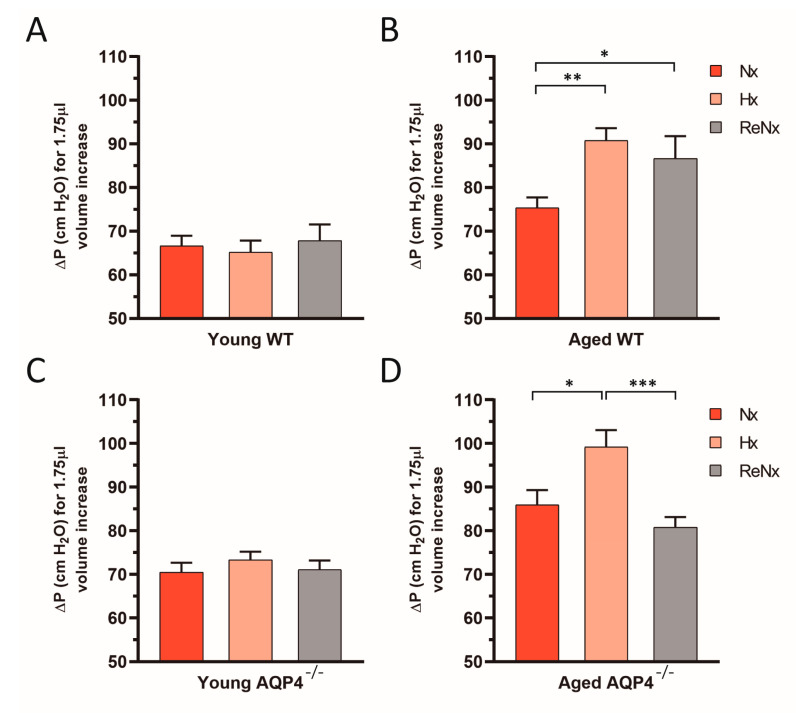
Alterations in the compliance of the cerebral ventricular system after re-normoxia. Quantitative analysis of the increment of IVP (ΔP) reached in 15 s after the infusion of 1.75 µL of artificial CSF for each experimental group. (**A**) Young wt, (**B**) aged wt, (**C**) young AQP4^−/−^, (**D**) aged AQP4^−/−^. Note that ventricular compliance is inversely proportional to ΔP, so the compliance of the ventricular system remains diminished in aged wt mice after the ReNx period but is recovered in aged AQP4^−/−^ animals. Data are represented as mean ± SEM. Number of animals analyzed for each experimental condition: Young-wt, Nx (*n* = 12); Hx (*n* = 7); ReNx (*n* = 6). Aged-wt, Nx (*n* = 8); Hx (*n* = 7); ReNx (*n* = 5). Young-AQP4^−/−^, Nx (*n* = 10); Hx (*n* = 8); ReNx (*n* = 8). Aged-AQP4^−/−^, Nx (*n* = 8); Hx (*n* = 6); ReNx (*n* = 8). * *p* < 0.05; ** *p* < 0.01; *** *p* < 0.001.

As for the observed CSF drainage capacity, young wt and AQP4^−/−^ mice did not show any alteration of the ventricular distensibility capacity under the various experimental conditions analyzed (Figure 4A,C). As we previously reported [19], however, Hx reduces the distensibility of the ventricular system in aged animals, both in wt mice and AQP4^−/−^ mice (Figure 4B,D). Interestingly, in the aged wt mice, which developed ventriculomegaly after Hx, the ventricular compliance remained diminished after the re-normoxic period (Figure 4B). Nevertheless, in the aged AQP4^−/−^ mice, which were protected from Hx-induced ventriculomegaly, the ventricular compliance was totally recovered after the ReNx period.

**Figure 5:**
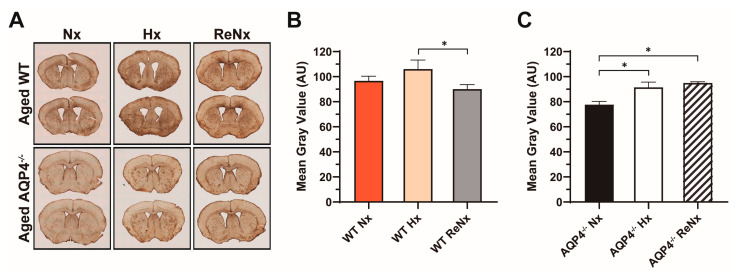
Astroglial response to hypoxia and re-normoxia of aged wt and AQP4^−/−^ mice. (**A**) Images, after GFAP immunohistochemistry, of coronal brain sections from aged wt and AQP4^−/−^ mice subjected to the experimental conditions indicated (Nx: normoxia; Hx: hypoxia; ReNx: re-normoxia). (**B**) Analysis of GFAP optical density of aged wt and AQP4^−/−^ mice in Nx. (**C**) Quantification of the astroglial response, by GFAP optical density measurements, to Hx and ReNx in aged wt and AQP4^−/−^ mice. Data are expressed as fold changes of GFAP labeling in either condition, Hx or ReNx, with respect to their own normoxic controls. Levels of GFAP in Nx for aged wt or AQP4^−/−^ mice were taken as 100% and are represented as dashed lines in each graph. Data are presented as mean ± SEM, and the number of animals tested for each experimental condition was *n* = 3, * *p* < 0.05.

To explore whether the different recovery response (ReNx) of the ventricular distensibility observed between aged wt and AQP4^−/−^ mice could be related to different levels of astrogliosis in these experimental groups, we analyzed the glial fibrillary acid protein (GFAP) expression level, as a standardized marker of astrogliosis, in aged wt and AQP4^−/−^ mice under our three oxygenation conditions: Nx, Hx, and ReNx (Figure 5). The first and most outstanding observation is that GFAP expression levels in the control-normoxic situation were considerably higher in the brain parenchyma of aged wt than in aged AQP4^−/−^ mice (Figure 5A,B). We analyzed the changes in GFAP expression induced by the Hx and ReNx treatments in both aged wt and AQP4^−/−^ mice, with respect to their own normoxic controls. In the case of aged wt mice, Hx induced a slight, but not significant, increase in GFAP expression, which returned to levels similar to those observed in normoxic controls after the ReNx period (Figure 5A,C). Conversely, in the aged AQP4^−/−^ mice, the hypoxic treatment produced a significant increment in GFAP expression levels that remained elevated after ReNx (Figure 5A,C) treatment. These data suggest a different astroglial response to Hx and ReNx in aged AQP4^−/−^ mice with respect to aged wt mice.

### 2.4. Repetitive Hypoxic Events Lead to Permanent and Irreversible Ventriculomegaly

**Figure 6:**
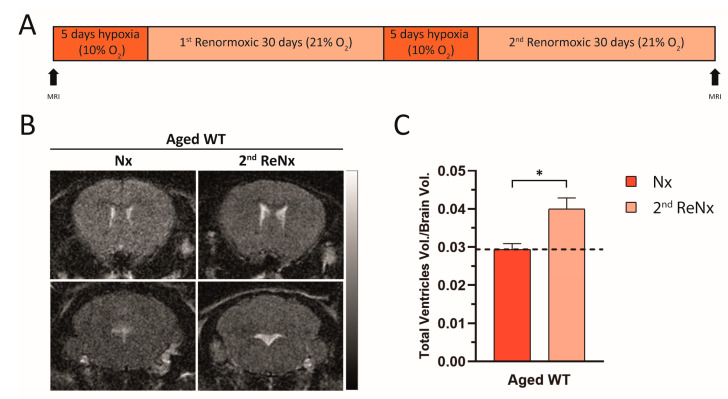
Consecutive hypoxic events produce permanent ventriculomegaly. (**A**) Schedule showing the experimental protocol. Briefly, aged wt mice were subjected to two consecutive cycles of Hx-ReNx, in which the Hx period consisted of 5 days at 9% O_2_ and the ReNx period consisted of 30 days at 21% O_2_. (**B**) Representative images of MRI coronal sections at the two time points analyzed, before the start of the Hx-ReNx cycles (Nx) and at the end of the treatment (second ReNx). The gradient scale of T2-weighted MRI indicates the liquid present in the tissue, ranging from bright (high water content) to dark (absence of water). (**C**) Quantification of brain ventricular volume under the conditions described in (**A**). Data are represented as mean ± SEM, The number of animals tested was *n* = 4, * *p* < 0.05.

Given that in aged wt mice ReNx treatment only produces a partial recovery of the severe ventriculomegaly induced by Hx, and CSF drainage and ventricular distensibility are permanently diminished, we investigated whether a second cycle of Hx-ReNx would lead to a poorer hydrocephalic state in the aged wt animals. We subjected aged wt mice to two cycles of Hx-ReNx (see scheme in Figure 6A). Given the difficulties of this experiment, we evaluated the animal’s ventricle size by MRI previous to initiating the Hx-ReNx cycles, in Nx, and at the end of the second ReNx period. As shown in Figure 6B,C, the ventriculomegaly situation obtained after the second hypoxic stimulus persisted, despite having returned to the ReNx situation. The ventricular volume after the second 30 days in ReNx was significantly different from the initial value in Nx (Figure 6C). The permanent deterioration in CSF drainage and ventricular compliance in the aged wt animals already established after the first hypoxic stimulus, together with the ventricular CSF overload produced in the second hypoxic event, probably triggered a severe and irreversible ventriculomegaly.

## 3. Discussion

The participation of the principal cerebral AQPs, choroidal-AQP1, and astroependimal-AQP4, in fundamental aspects of CSF homeostasis constitute a currently accepted body of knowledge [6,11]. Both proteins play an important role in the formation and drainage of the CSF, hence contributing to intraventricular pressure. They also appear to participate in determining structural functions related to the distensibility capacity of the ventricular system [5,19]. A previous study using aged animals exposed to Hx showed the development of a hydrocephalic condition that reproduced fundamental pathophysiological features of patients with iNPH, such as ventriculomegaly, elevation of intracranial pressure, cognitive impairment, and reduced CSF drainage and ventricular distensibility, with clear participation of AQP4 in the CSF ventricular overload during the hypoxic event [19]. In the present study, we explored in the same experimental model whether these alterations associated with the hydrocephalic state are permanent or could be reverted by ReNx.

As previously reported [19], Hx produced a significant increment in the total ventricle volume, either in young (~15% larger) or aged (~40% larger) wt mice. We also showed that reexposing the animals to Nx for 30 days (ReNx) led to ventricle volumes returning to values similar to those measured in the normoxic control situation. Similarly, the IVP records showed a parallel trend, reestablishing values similar to those measured prior to the hypoxic stimulus. Thus, when returning to a normal state of oxygenation, cerebral blood perfusion will be reduced, and CSF and IVP diminishes as well, as evidenced by our data. In the AQP4^−/−^ mice, both young and aged, the hypoxic treatment did not produce ventriculomegaly or alterations in the IVP values. Accordingly, in these AQP4 mutants, the ReNx treatment did not alter either the ventricle size or the IVP values. Furthermore, the results from the behavioral test revealed that even in the aged wt mice, normal cognitive function was reestablished after the ReNx period, which could indicate that once the ventriculomegaly situation is removed, the cognitive impairment also disappears. However, the CSF drainage capacity and the ventricular compliance deteriorated similarly in both wt and AQP4^−/−^ aged animals when they were subjected to Hx. Thus, the alteration of these two parameters during a hypoxic stimulus appears to be associated with the aging process, rather than with the presence of AQP4. However, after ReNx, the reversion of these parameters to initial values only took place in the aged AQP4^−/−^ mice, whereas they remained altered in the aged wt animals. In the aged wt animals, in which Hx produces ventriculomegaly, the alterations in CSF drainage and ventricular compliance were not recovered after ReNx. In contrast, in the aged AQP4^−/−^ mice, which did not develop ventriculomegaly in Hx, both parameters returned to normoxic control values in ReNx. Therefore, definitive damage to the CSF evacuation system occurs in the aged wt animal and is dependent on AQP4 expression. These results suggest that the recovery of these parameters could be conditioned by the occurrence, or not in the case of AQP4^−/−^ aged mice, of a severe ventriculomegaly.

Apart from the fact that irreversible deterioration of the CSF outflow and ventricular distensibility only occur when a severe ventriculomegaly is developed, as occurred in the aged wt animals, alternative explanations for these chronic alterations could include cellular changes in astrocytes linked to AQP4 expression, such as astrogliosis [19,23,24,25,26]. To explore this hypothesis, we studied GFAP expression, as a standard marker of astrogliosis, in aged wt and AQP4^−/−^ mice subjected to Hx or ReNx. Importantly, the aged AQP4^−/−^ mice showed lower levels of astrogliosis than the wt controls in normoxic conditions. In addition, the Hx/ReNx treatment produced a different astroglial response between the aged wt and AQP4^−/−^ mice. In the aged wt mice, the Hx/ReNx treatment induced a slight and transient astrogliosis. Conversely, the same treatment produced a significant and permanent astrogliosis in the aged AQP4^−/−^ mice. Although our data about astrogliosis cannot completely explain the difference in ventricular distensibility observed between the aged wt and AQP4^−/−^ mice in ReNx, they highlight the relevance of AQP4 in the process of astrogliosis linked to aging and hypoxia. It would be interesting to more deeply explore the specific role of AQP4 and other structural partners of the astrocyte-extracellular matrix-endothelial complex (i.e., dystroglycan, alpha-syntrophin, dystrobrevin, etc) in the astroglial response and their potential relationship with ventricular compliance.

Employing different cellular markers, Hannock et al. [23] described molecularly distinct compartments surrounding different vessel types and provided a comprehensive characterization of the arachnoid and pial compartments and their connection to CNS vessels and perivascular pathways. Staining for AQP4, GFAP, and plectin permitted these authors to identify various populations of astrocytes and observe how they differentially connect to distinct laminin proteins depending on the basal membrane with which they connect [23]. We hypothesize that a lack of AQP4 expression would potentially alter the protein expression profile of astrocytes and hence their extracellular contacts, altering at the end the properties of the basal membrane surrounding the CSF compartments.

Another important result presented here is that two repetitive hypoxic episodes produce a permanent and irreversible ventriculomegaly and likely permanent cognitive deterioration. In terms of the etiology and development of iNPH, repetitive Hx events throughout life might lead to a poorer prognosis in the course of the disease. Despite being defined as a pathology of unknown etiology, multiple factors are frequently associated with the establishment of iNPH. Population studies have shown that almost 25% of patients diagnosed with iNPH have one or more cardiovascular risk factors, including high blood pressure, hyperlipidemia, diabetes, obesity, sedentary lifestyle, and peripheral and cerebral vascular diseases, as well as psychosocial factors [27,28]. At the same time, it has been stated that up to 50% of the population older than 65 years of age has been exposed to, or had at least one of, the following respiratory risk factors: obstructive sleep apnea/hypopnea syndrome, chronic obstructive pulmonary disease, chronic smoking, and exposure to fumes, dust, and environmental pollutants [29,30]. Sleep-disorder breathing as observed in obstructive sleep apnea has been indicated to affect the proper circulation of interstitial CSF into the glymphatic circulation contributing to iNPH [29].

Our data show, for the first time, that hypoxic events promote irreversible damage of the CSF drainage capacity and ventricular distensibility in aged cerebral tissue, these anomalies being dependent on the AQP4 expression. These data, together with a recent report linking AQP4 to the severity of traumatic brain edema [31], make this protein a potential drug target in neurological disorders associated with water homeostasis. Additionally, the permanent alterations in CSF drainage and ventricular compliance together with an increase in CSF formation during subsequent hypoxic events would explain the development of chronic ventriculomegaly as occurs in patients with iNPH. Additionally, exposure throughout life to any of the aforementioned conditions is likely to generate recurrent episodes of Hx that produce an even greater deterioration of these parameters, thus leading to a hydrocephalic status with a major and irreversible ventriculomegaly.

## 4. Materials and Methods

### 4.1. Animal Care and Hypoxic Treatments

AQP4^−/−^ mice and wt littermates were genotyped as previously indicated [32]. The mice were housed under a controlled temperature (22 ± 1 °C) in a 12 h light/dark cycle, with ad libitum access to food and water. Young mice were considered as 2–4 months old, and aged as >14 months old. The animals were maintained either in normoxic conditions (21% O_2_; Nx), exposed to hypoxia (5 days at 9% O_2_, Hx), or reexposed to normoxia after hypoxic treatment for 30 days (ReNx), using a hermetically sealed chamber (Coy Laboratory Products, Inc., Grass Lake, MI, USA) as previously described [33], with continuous monitoring and control of gas concentrations, temperature, and humidity. For euthanasia, the mice were anesthetized with a combination of 100 mg/kg ketamine (Pfizer) and 10 mg/kg xylazine (Bayer). All experiments were performed in accordance with the European Directive 2010/63/EU and the Spanish RD/53/2013 on the protection of animals used for scientific purposes. The study was approved by the Animal Research Committee of Virgen del Rocío University Hospital (26/01/2017/017; University of Seville).

### 4.2. Magnetic Resonance Imaging

An MRI of the mouse brain was taken to assess ventricle size as an indicator of CSF production. For this, the mice were anesthetized using 0.5–2.5% sevoflurane with spontaneous breathing. MRI studies were performed using an ICON 1 Tesla system (Bruker, Billerica, MA, USA), provided with a mouse body radiofrequency coil, available at the animal facilities of the institute (IBiS). Ventricular volumes were estimated from T2-weighted 3D rapid acquisition with relaxation enhancement sequences of coronal brain sections (repetition time, 95 ms; echo time, 3250 ms; plane resolution, 0.188 × 0.188 × 0.563 mm; thickness, 0.5 mm; rare factor, 8; and 32 slices). The images were then analyzed in a computer loaded with VIRTUE software (Diagnosoft, Palo Alto, CA, USA) and ImageJ 1.45 software (Wayne Rasband, National Institutes of Health).

### 4.3. Intraventricular Pressure Measurements

IVP measurements were performed as previously described [5,19]. Briefly, mice were anesthetized with 100 mg/kg ketamine (Pfizer, New York, NY, USA) and 10 mg/kg xylazine (Bayer, Leverkusen, Germany) and immobilized, in prone position, in a stereotaxic device (Stoelting, St. Louis, MO, USA). A 34-gauge micropipette (Hamilton, Reno, NV, USA) filled with artificial CSF (aCSF) was placed in the parietal cortex above the lateral ventricle (from bregma in mm: anteroposterior, −0.2; lateral, +1.0; and ventral, −1.0). Then, the micropipette was inserted into the lateral ventricle through the cerebral cortex and a syringe pump (KD Scientific, Holliston, MI, USA) was used to perfuse the aCSF into the ventricles at an infusion rate of 0.3 μL/min. The micropipette was connected to a pressure transducer (TSD104A; Biopac Systems, Goleta, CA, USA) using noncompliant tubing interfaced to a recording system (model MP150; Biopac Systems, Goleta, CA, USA). Once the pipette entered the brain parenchyma, the pressure gradually increased; when the pressure was >40 cm H_2_O, the infusion was stopped and the pipette was slowly advanced. The pressure dropped promptly when the pipette tip reached the lateral ventricle. Then, IVP was continuously monitored and recorded.

### 4.4. Novel Object Recognition Testing

Novel object recognition tests were performed as previously described [19]. Before starting the behavioral test, the mice were left for 10 min to explore the arena box (50 × 50 × 40 cm). Then, during the sample trial, the mice were placed into the arena with two identical objects and the behavior of the animal was video recorded from a suspended camera for 10 min. After this, the mouse was removed from the box and placed back into its home cage and the arena was cleaned. After 3 h, the mice were tested again in the same arena, but with one of the two familiar items randomly replaced with a novel object (with similar discrimination index), and video recorded again for 10 min. The interactions of the animal with the object were analyzed with Novel Object Recognition Biobserve software (Biobserve GmbH, Bonn, Germany).

### 4.5. CSF Outflow Dynamics and Ventricular Compliance Measurements

Measurements of CSF outflow and ventricular compliance were performed as previously reported [5,19], employing the constant-rate infusion method reported originally by Oshio et al. [22]. Briefly, a micropipette was placed in the lateral ventricle as described above, and IVP was recorded during continuous infusion of aCSF into the lateral ventricle at rates of 0.5, 1.5, 3, 7, and 14 μL/min. At each rate of infusion, the aCSF input was maintained until a steady-state IVP was recorded, resulting in a step-wise increase in IVP according to increments in the perfusion rate. The CSF outflow resistance was obtained by the slope of the linear regression achieved by plotting the different infusion rate used vs the attained IVP for each. To normalize the IVP values, basal IVP value (obtained without aCSF infusion) were subtracted from the IVP values obtained after aCSF infusion at each rate. For the various experimental animals and treatments, the ventricular distensibility was calculated by measuring the increase in IVP produced by the infusion of 1.75 μL of aCSF at a rate of 14 μL/min.

### 4.6. Histological Analyses and Densitometry

The mice were subjected to transcardial perfusion with 50 mL of phosphate-buffered saline (PBS, Sigma, Kawasaki, Japan) and 50 mL of 4% paraformaldehyde (Sigma, Kawasaki, Japan) in PBS. After that, the brains were immediately removed and fixed with 4% paraformaldehyde in PBS overnight at 4 °C. After fixation, the brains were cryoprotected in 30% sucrose (Sigma, Kawasaki, Japan) in PBS and included in Optimum Cutting Temperature compound (Tissue-Tek, Torrance, CA, USA). Coronal sections (30-μm thickness) were cut on a cryostat (Leica, Wetzlar, Germany). GFAP immunohistochemistry was performed as previously described [34], using polyclonal anti-GFAP (1:500; Dako, Beijing, China) and a secondary peroxidase-conjugated antibody kit (Dako, Beijing, China). Images were taken with a light-transmitted microscope and a digital camera (Olympus, BX61-UCMAD3, Shinjuku, Japan) using New CASTTM system (Visiopharm, Hørsholm, Denmark) software.

To estimate astrogliosis, the optical density of GFAP^+^ staining was measured from digitized pictures using NIH Image software (ImageJ) as previously described [33], analyzing 10 slices per animal, covering the periventricular brain system, including lateral ventricles, dorsal and ventral sides of third ventricles, from +1.94 mm to −2.70 mm rostro-caudal relative to Bregma according to the Franklin and Paxinos mouse brain stereotaxic atlas [35].

### 4.7. Statistical Analysis

The data are presented as mean ± standard error of the mean (SEM). Each experimental group included 3–12 mice; the specific numbers (*n*) are shown in each figure. In all cases, the data were tested for normality (Kolmogorov–Smirnov test) and equal variance test. When these properties were confirmed, an analysis of variance was performed with Bonferroni post hoc analysis (for multiple group comparisons) or Student’s *t*-test (for two-group comparisons); otherwise, the nonparametric Kruskal–Wallis H test (for multiple comparisons) or Mann–Whitney U test (for two-group comparisons) were used. All statistical analyses were conducted using IBM SPSS Statistics for Windows, Version 20 or Prism8.

## Data Availability

All important data is included in the manuscript.
